# INFLUENCE OF ECONOMIC WELL-BEING ON HEALTH, AND MODERATING EFFECT OF SOCIAL ENGAGEMENT AMONG OLDER CAMBODIANS

**DOI:** 10.1093/geroni/igz038.014

**Published:** 2019-11-08

**Authors:** Kakada Kuy, Sojung Park

**Affiliations:** 1 WASHINGTON UNIVERSITY IN ST LOUIS, St. Louis, United States; 2 Brown School, Washington University in St. Louis, St. Louis, Missouri, United States

## Abstract

Similar to other post-conflict nations in Southeast Asia, impacts of civil wars and violence, coupled with the present poverty, place older Cambodians in vulnerable health conditions. Older Cambodians have limited access to basic healthcare and decent living conditions. To date, little research has been conducted to understand their health and related determinants. This study aimed to examine the influence of economic wellbeing and the moderating effect of social engagement on the physical health of older Cambodians. Data came from Survey of Elderly Cambodia (2004), the only existing nationally representative dataset of older Cambodians age 60 and above (N=1,273). Economic wellbeing was measured in two dimensions: (1) subjective economic wellbeing with three indicators of self-rated economic satisfaction and (2) objective economic wellbeing with household item possession and current housing conditions. Social engagement was examined with four types of engagement in the community. For physical health, the number of health complaints was examined. Results of Poisson regression showed objective economic wellbeing, such as access to basic household items and decent housing conditions, was significantly related to physical health (p < .001). However, we found no role of social engagement in the association between the subjective economic wellbeing and health. Furthermore, social engagement has a significant moderating effect on the association between objective economic wellbeing and physical health (p < .001), controlling for all covariates. For anti-poverty and health policy development for older Cambodians, a promising intervention effort may focus on the ways to facilitate social engagement in later years.

